# Clinical characteristics and long-term follow-up of seven cases of anti-GABABR encephalitis in patients of Han Chinese descent

**DOI:** 10.1007/s10072-019-04095-9

**Published:** 2019-10-28

**Authors:** Wei Zeng, Liming Cao, Jinou Zheng, Lu Yu

**Affiliations:** 1 Department of Neurology, Liuzhou People’s Hospital, Wenchang Road, Chengzhong District, Liuzhou City, 545000 China; 2 Department of Neurology, The 3rd Affiliated Hospital of Shenzhen University, 47 Friendship Road, Luohu District, Shenzhen City, 518000 China; 3 Department of Neurology, First Affiliated Hospital of Guangxi Medical University, 6 Shuangyong Road, Qingxiu District, Nanning City, 530021 China

**Keywords:** anti-gamma-aminobutyric acid B receptor encephalitis, clinical features, treatment, prognosis

## Abstract

**Objective:**

To improve the diagnosis and treatment of anti-GABAB receptor (anti-GABABR) encephalitis and prevent misdiagnosis or non-diagnosis.

**Methods:**

We retrospectively examined the chief clinical manifestations, auxiliary examination results, treatment strategies, treatment efficacy, and long-term follow-up results of seven consecutive patients with anti-GABABR encephalitis.

**Results:**

Epileptic seizures were the first symptom in 100% of the patients; 85.7% had memory deficit in the hospital, 42.8% had residual symptoms of cognitive impairment at discharge, and 28.6% had cognitive impairment at the end of follow-up; 71.4% of the patients had psychosis in the hospital, 57.1% had residual symptoms of psychosis at discharge, and 14.3% still had psychosis at the end of follow-up. However, the clinical symptoms (psychiatric disorders, cognitive decline) and signs (consciousness disturbance) at onset and after follow-up were not significantly different (*P* > 0.05). In 71.4% of the patients, anti-GABABR antibody serum levels were higher than those in the cerebrospinal fluid (especially in patients with lung cancer). Magnetic resonance imaging in 71.4% of patients indicated that the marginal lobe demonstrated encephalitis lesions. The average modified Rankin Scale score (2.0 **±** 2.31) at follow-up was significantly better than that (3.86 **±** 0.90) at the time of admission (*P* < 0.05).

**Conclusion:**

The clinical characteristics of anti-GABABR encephalitis were refractory epilepsy, psychiatric disorders, and cognitive impairment. Multiple antiepileptic drugs are crucial for the treatment of intractable epilepsy. Clinicians should eliminate the possibility of small-cell lung cancer in patients with high anti-GABABR antibody levels. Early active immunotherapy is effective, and the long-term prognosis is good for patients without tumors.

## Introduction

Anti-GABAB receptor (anti-GABABR) encephalitis is a newly reported form of autoimmune encephalitis associated with anti-neuron surface antigen antibodies [1]. The rate of misdiagnosis is high, since only a few cases have been reported to date and the clinical manifestations and prognosis of anti-GABABR encephalitis have yet to be investigated systematically [2]. We analyzed the clinical manifestations, auxiliary examination results, treatment strategies, and long-term prognoses in seven consecutive patients with anti-GABABR encephalitis, to improve the diagnosis and treatment of anti-GABABR encephalitis.

## Clinical materials and methods

### Patient characteristics

The study included seven consecutive patients (women: 3, men: 4; age: 44.7 ± 14.09 years, range: 27–64 years) with anti-GABABR encephalitis, who were treated at the Department of Neurology at the First Affiliated Hospital of Guangxi Medical University (China). The duration of the follow-up period was 16.14 ± 4.41 months and ranged from 10 months to 2 years. No patient had a relevant family history of genetic abnormalities, immunodeficiency history, or developmental abnormalities.

### Methods

#### Diagnostic criteria

All patients met the following diagnostic criteria [3] for anti-GABABR encephalitis:

1. acute or subacute onset, progressive aggravation; (2) clinical symptoms in accordance with the characteristics of marginal encephalitis; (3) slightly elevated lymphocyte levels and/or normal white blood cell (WBC) count in the cerebrospinal fluid (CSF); (4) presence of anti-GABABR antibodies in the serum and/or CSF; abnormal signals in the unilateral/bilateral medial temporal lobe or an absence of lesions on brain magnetic resonance imaging (MRI); and (5) abnormal electroencephalogram (EEG) findings. Patients with lesions suggestive of herpes simplex encephalitis, toxic encephalopathy, acute disseminated encephalomyelitis, or multiple sclerosis were excluded.

#### Experimental detection and treatment methods

Serum and CSF samples were collected and sent to Guangdong Jinyu Inspection Company (Guangzhou, China) for commercial testing to detect the following autoimmune encephalitis antibodies: anti-GABABR antibodies, anti-glutamate receptor (including NMDA, AMPA1, and AMPA 2) IgG antibodies, anti-leucine-rich glioma-inactivated protein 1 IgG antibodies, and anti-contact protein associated protein 2 IgG antibodies. The antibodies were detected using an indirect immunofluorescence test, which is widely accepted as the most suitable method. All patients received corticosteroid pulse therapy (methylprednisolone 1 g/day for 5 days, 0.5 g/day for 5 days, 0.25 g/day for 5 days, 0.125 g/day for 5 days, and subsequent oral administration), which was accompanied by intravenous gamma globulin treatment (0.4 g/kg/day for 5 days) in 28.5% of patients. Rehabilitation efficacy was assessed according to the modified Rankin Scale (mRS) scores, as a measure of global disability [4].

### Statistical analysis

We retrospectively analyzed the mode of onset, initial symptoms, main clinical manifestations, auxiliary examination results, treatment strategies, and long-term prognosis for each patient. The data were analyzed using the SAS 9.3 statistical software. The measured data exhibited normal distribution and were expressed as mean ± standard deviation. The Student *t* test, for paired data, was used to compare the respective mRS scores at follow-up and admission. The clinical symptoms and signs at onset and after follow-up were compared using the exact probability method.

## Results

**Fig. 1:**
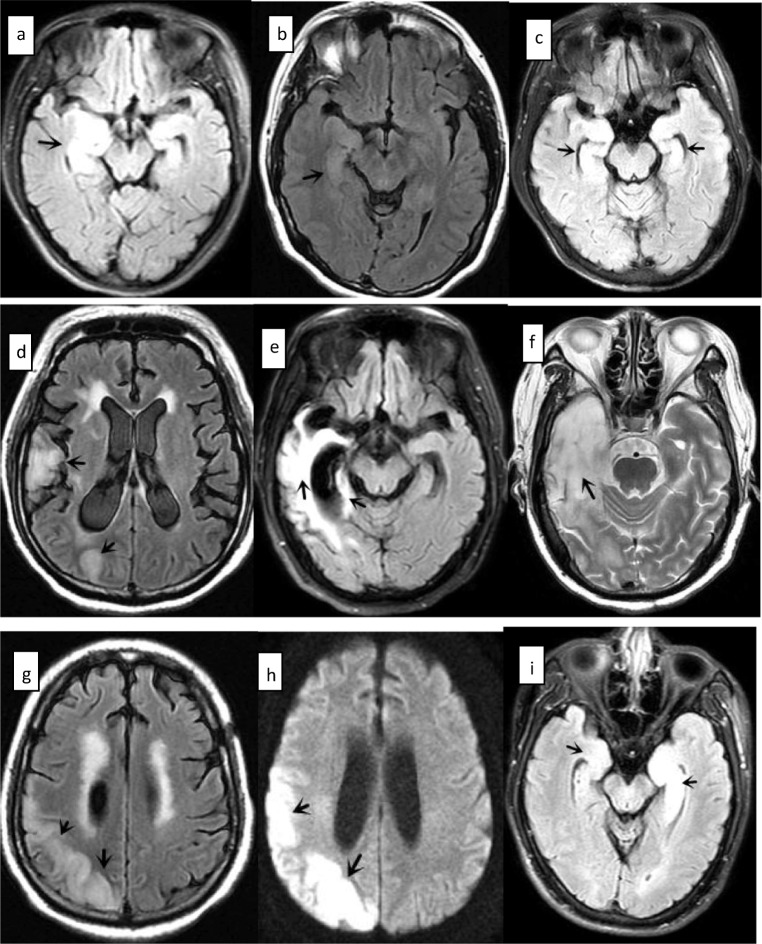
Patient 1: **a** T2-FLAIR showing hyperintensity in the right hippocampus, parahippocampal gyrus, and amygdala. Patient 2: **b** T2-FLAIR showing hyperintense lesions in the right temporal lobe. Patient 3: **c** T2-FLAIR showed hyperintensities in the hippocampus, bilaterally, and in the medial temporal lobe. Patient 5: **d** T2-FLAIR showing abnormal signals in the right temporal lobe. **e** T2-FLAIR showing abnormal signals in the right temporal lobe and hippocampus. **f** T2-weighted sequence showing hyperintense lesions in the right temporal lobe. **g** T2-FLAIR showing hyperintensity in the right temporal occipital lobes. **h** Diffusion-weighted imaging sequence showing hyperintensity in the right temporal and occipital lobes. The lesions described above are marked with arrows. Patient 7: **i** T2-FLAIR showing abnormal signal shadows in the hippocampus, bilaterally (more obvious on the left). Patient 4 and patient 6 did not show any encephalitis lesions on magnetic resonance imaging. FLAIR: fluid-attenuated inversion recovery

The clinical symptoms, auxiliary examination results, treatment strategies, and long-term follow-up results in patients with anti-GABABR encephalitis are presented in Table 1. No symptoms of infection, including cold, diarrhea, fever, or vomiting, were observed in 85.7% of patients prior to onset. Epileptic seizures were the first symptom in 100% of patients. Memory deficits were observed in 85.7% of patients in the hospital, 42.8% had residual symptoms of cognitive impairment at discharge, and 28.6% still had cognitive impairment at the end of the follow-up period. Furthermore, 71.4% of patients had psychosis in the hospital, 57.1% had residual symptoms of psychosis at discharge, and 14.3% still had psychosis at the end of the follow-up period. No significant difference was observed in the clinical symptoms (psychiatric disorders and cognitive decline) and signs (consciousness disturbance) at onset and after follow-up (*P* > 0.05). The CSF WBC count and protein levels were both slightly elevated in 14.3% of patients. Serum anti-GABABR antibody levels were higher than those in the CSF in 71.4% of patients, especially in the two patients with lung cancer. MRI demonstrated encephalitis lesions in the marginal lobe of 71.4% of patients. Low-intensity or equisignal lesions on T1-weighted imaging and high-intensity lesions were observed on T2-weighted imaging. None of the lesions showed significant enhancement (Fig. 1). The average mRS score at follow-up (2.0 **±** 2.31) was significantly better than that (3.86 **±** 0.90) at admission (*P* < 0.05). No recurrence of epilepsy or encephalitis was observed in any patient during the follow-up period.

**Table 1:** Clinical symptoms and long-term follow-up results of seven patients with anti-GABABR encephalitis

Assessment	Patient 1	Patient 2	Patient 3	Patient 4	Patient 5	Patient 6	Patient 7
Prodromal symptoms^a^ (14.3%)	No	Yes	No	No	No	No	No
Psychiatric disorders (5/7, 71.4%)	No	Yes	Yes	Yes	Yes	Yes	No
Cognitive decline (6/7, 85.7%)	Yes	Yes	Yes	No	Yes	Yes	Yes
Consciousness disturbance (2/7, 28.6%)	No	No	Yes	No	No	No	Yes
Epilepsy (100%)	Yes	Yes	Yes	Yes	Yes	Yes	Yes
Involuntary movement (2/7, 28.6%)	No	No	No	Yes	No	No	Yes
CSF pressure (mmH_2_O)	–	–	220	–	210	–	–
WBC in CSF^b^	–	–	–	67 × 10^6^/L	–	–	–
CSF protein^c^	–	–	–	–	594 mg/L	–	–
EEG	Epileptic waves	Moderate abnormality	Mild or moderate abnormality	---	epileptic waves	Epileptic waves	Moderate abnormality
Lesions on brain MRI	Right hippocampus and amygdala	Right temporal lobe	Bilateral hippocampus and medial temporal lobe	No lesion	Right temporal lobe and occipital lobe	No lesion	Bilateral hippocampus
Tumors	–	Cervical cancer	Lung cancer	Lung cancer	–	–	–
Serum antibody titer	1:100	1:10	1:320	1:320	1:10	1:10	1:100
CSF antibody titer	1:100	1:32	1:10	1:32	Negative	Negative	1:10
mRS score at admission (3.86 ± 0.90)	3	4	5	5	3	3	4
Corticosteroid pulse therapy	Yes	Yes	Yes	Yes	Yes	Yes	Yes
IVIg	No	No	No	No	No	Yes	Yes
AED treatment in hospital	CBZ, BDZ	CBZ, BDZ	TPM, VPA	VPA, BDZ	VPA, LEV	PB, OXC, TPM	VPA, BDZ
Residual symptoms at discharge	Hypophrenia	Hypophrenia and psychosis	Psychosis	Psychosis	Hypophrenia	Psychosis	No
Follow-up period	19 months	16 months	14 months	10 months	14 months	16 months	24 months
Consciousness (0%)	Clear	Clear	Clear	Clear	Clear	Clear	Clear
Cognitive function (2/7, 28.6%)	Normal	Normal	Normal	Normal	Memory deficits	Normal	Memory deficits
Psychiatric disorders (1/7, 14.3%)	No	No	No	Yes	No	No	No
mRS score at follow-up (2.0 ± 2.31	0	0	5	5	3	0	1
Prednisone treatment after discharge	1 month	3 months	< 1 month	< 10 months	< 1 month	3 months	6 months
Azathioprine treatment after discharge	1 month	1 year	2 months	< 10 months	< 1 month	1 year	1 year
AED treatment after discharge	CBZ × 1 month	CBZ × 3 months	TPM VPA < 1 month	VPA × 10 months	VPA, LEV × 14 months	PB, OXC, TPM × 16 months	VPA × 6 months

Note: The modified Rankin scores at baseline/admission and after follow-up are denoted as the mean ± standard deviation (*t* = 3.240, *P* = 0.018 < 0.05). The clinical symptoms (psychiatric disorders, cognitive decline) and signs (consciousness disturbance) at onset and after follow-up did not exhibit a statistically significant difference (total *P* > 0.05). “–” stands for normal

*CSF* cerebrospinal fluid, *WBC* white blood cell, *EEG* electroencephalography, *MRI* magnetic resonance imaging, *IVIg* intravenous immunoglobulin, *mRs* modified Rankin scale, *AED* antiepileptic drug, *CBZ* carbamazepine, *LEV* levetiracetam, *OXC* oxcarbazepine, *PB* phenobarbitone, *VPA* valproic acid, *BDZ* benzodiazepines, *TPM* topiramate, *SD* standard deviation

^a^Prodromal symptoms refer to headache, vomiting, fever, and diarrhea

^b^Normal range of CSF WBC count: 0–8 × 10^6^/L

^c^Normal range of CSF proteins: 150–450 mg/L

## Discussion

### Clinical manifestations

Viral infection is among the principal causes of autoimmune encephalitis [5]. However, only a few patients in our study had prodromal symptoms of infection; infection was not a trigger for the onset in most patients. Epilepsy is a prominent and critical clinical manifestation of anti-GABABR encephalitis, and epilepsy or refractory epilepsy is often the first symptom [6]. In a previous study by Guan et al., 17 of the 18 patients presented with new refractory epilepsy or status epilepticus [7], while all the patients in our study had epilepsy. Herpes simplex encephalitis is perhaps most frequently associated with epilepsy, of the types of sporadic viral encephalitis, with an incidence of about 50% in cases of combined epilepsy [8]. The risk of seizures in population-based cohorts of survivors of central nervous system infections is between 6.8 and 8.3% [9]. Three-quarters of patients with anti-GABABR antibodies developed refractory seizures [10]. In short, these results indicate that the incidence of epilepsy in anti-GABABR encephalitis was significantly higher than that in viral, infectious, or autoimmune encephalitis. High levels of anti-GABABR antibodies are associated with seizures, refractory status epilepticus, or both [11]. Anti-GABABR encephalitis epilepsy occurs due to an immune reaction to neuronal elements, driven by an underlying malignancy. Our study showed that memory deficit is common in these patients and that complete recovery is difficult, which may be closely related to damage to the marginal lobe. Psychiatric disorders are common (64.7%) [7] in patients with anti-GABABR encephalitis, and this result is roughly in line with our finding. Mental disorders are also common symptoms that tend to persist until the patient is discharged. No statistically significant difference was observed (*P* > 0.05) between the clinical symptoms (psychiatric disorders, cognitive decline) and signs (consciousness disturbance) at onset and after follow-up, which may be attributed to the small sample size.

### Auxiliary examination results

Non-specific inflammatory changes, including slight increases in lymphocyte count and protein levels in the CSF, were observed in our patients. These were similar to the observations in viral encephalitis. Our results suggest that anti-GABABR encephalitis cannot be excluded in patients without lesions on MRI. Most EEG results revealed moderate to severe abnormalities, such as increases in slow waves and epileptic waves. Routine EEG should be performed in patients with suspected anti-GABABR encephalitis. Our research suggests that anti-GABABR antibodies are first produced in the blood, and subsequently enter the CSF by crossing the blood-brain barrier. Serum antibody levels should be evaluated to avoid a misdiagnosis, if patients test negative for anti-GABABR antibodies in the CSF. The pathogenesis of anti-GABABR encephalitis may be related to immune dysfunction caused by tumors. Small-cell lung cancer is associated with limbic encephalitis [2], and 50% of patients with anti-GABABR encephalitis were also diagnosed with small-cell lung cancer [12]. Therefore, patients with anti-GABABR encephalitis should be regularly screened for tumors (mainly small-cell lung cancer), especially when the serum antibody levels are significantly higher than those in the CSF.

### Treatment and long-term prognosis

Early immunotherapy is recommended for treatment of anti-GABABR encephalitis [3]. Early immune modulation may prevent serious potential consequences [13] and may result in a good prognosis [2], except for patients with malignant tumors. First-line immunotherapy includes corticosteroid pulse therapy, plasma exchange, and intravenous immunoglobulin therapy. We recommend the use of corticosteroid pulse therapy, because it is less expensive and easy to administer. Combined intravenous immunoglobulins may be effective, if corticosteroid pulse therapy is ineffective. Second-line immunotherapy includes cyclophosphamide and/or rituximab [14], which can be administered in case of suboptimal results with the first-line treatment. Our research showed that most patients exhibited some residual symptoms at discharge, with varying degrees of memory deficit, even at the end of the follow-up. Monotherapy was used in 54.1% of patients with epilepsy [15]. All the patients in our study required treatment with two or more antiepileptic drugs (AEDs), which were gradually reduced or even discontinued in several patients, during follow-up. Patients with autoimmune encephalitis have a high rate of seizure remission, and the long-term use of AEDs may be unnecessary to control seizures [10]. The long-term effects of active treatment were good, despite the difficulty in treating early epilepsy in anti-GABABR encephalitis.

As this observational study included a small number of patients, our findings should be interpreted with caution, and further studies are required to confirm the generalizability of our results.

## Conclusion

The clinical characteristics of anti-GABABR encephalitis are refractory epilepsy, psychiatric disorders, and cognitive impairment. Tests for anti-GABABR antibodies, brain MRI, and EEG should be performed as early as possible, and corticosteroid pulse therapy is the treatment of choice. If corticosteroid pulse therapy is unsuccessful, combined treatment with intravenous immunoglobulin may be effective. Patients should undergo screening for small-cell lung cancer (especially patients with high antibody titers). Early treatment with multiple AEDs is crucial for refractory epilepsy. Active immunotherapy may ensure a good long-term prognosis for patients without tumors.
